# Uncoupled Embryonic and Extra-Embryonic Tissues Compromise Blastocyst Development after Somatic Cell Nuclear Transfer

**DOI:** 10.1371/journal.pone.0038309

**Published:** 2012-06-06

**Authors:** Séverine A. Degrelle, Florence Jaffrezic, Evelyne Campion, Kim-Anh Lê Cao, Daniel Le Bourhis, Christophe Richard, Nathalie Rodde, Renaud Fleurot, Robin E. Everts, Jérôme Lecardonnel, Yvan Heyman, Xavier Vignon, Xiangzhong Yang, Xiuchun C. Tian, Harris A. Lewin, Jean-Paul Renard, Isabelle Hue

**Affiliations:** 1 INRA, UMR 1198 Biologie du Développement et Reproduction, Jouy-en-Josas, France; 2 ENVA, Maisons Alfort, France; 3 INRA, UMR1313, Génétique Animale et Biologie Intégrative, Jouy-en-Josas, France; 4 INRA, UR631, Station d’Amélioration Génétique des Animaux, Castanet, France; 5 UNCEIA, R&D Department, Maisons Alfort, France; 6 INRA, UE331 UCEA de Bressonvilliers, Leudeville, France; 7 Department of Animal Sciences, University of Illinois at Urbana-Champaign, Urbana, Illinois, United States of America; 8 INRA, Centre de Ressources Biologiques, Jouy-en-Josas, France; 9 Department of Animal Science and Center for Regenerative Biology, University of Connecticut, Storrs, Connecticut, United States of America; 10 Institute for Genomic Biology, University of Illinois at Urbana-Champaign, Urbana, Illinois, United States of America; Kanazawa University, Japan

## Abstract

Somatic cell nuclear transfer (SCNT) is the most efficient cell reprogramming technique available, especially when working with bovine species. Although SCNT blastocysts performed equally well or better than controls in the weeks following embryo transfer at Day 7, elongation and gastrulation defects were observed prior to implantation. To understand the developmental implications of embryonic/extra-embryonic interactions, the morphological and molecular features of elongating and gastrulating tissues were analysed. At Day 18, 30 SCNT conceptuses were compared to 20 controls (AI and IVP: 10 conceptuses each); one-half of the SCNT conceptuses appeared normal while the other half showed signs of atypical elongation and gastrulation. SCNT was also associated with a high incidence of discordance in embryonic and extra-embryonic patterns, as evidenced by morphological and molecular “uncoupling”. Elongation appeared to be secondarily affected; only 3 of 30 conceptuses had abnormally elongated shapes and there were very few differences in gene expression when they were compared to the controls. However, some of these differences could be linked to defects in microvilli formation or extracellular matrix composition and could thus impact extra-embryonic functions. In contrast to elongation, gastrulation stages included embryonic defects that likely affected the hypoblast, the epiblast, or the early stages of their differentiation. When taking into account SCNT conceptus somatic origin, i.e. the reprogramming efficiency of each bovine ear fibroblast (Low: 0029, Med: 7711, High: 5538), we found that embryonic abnormalities or severe embryonic/extra-embryonic uncoupling were more tightly correlated to embryo loss at implantation than were elongation defects. Alternatively, extra-embryonic differences between SCNT and control conceptuses at Day 18 were related to molecular plasticity (high efficiency/high plasticity) and subsequent pregnancy loss. Finally, because it alters re-differentiation processes *in vivo,* SCNT reprogramming highlights temporally and spatially restricted interactions among cells and tissues in a unique way.

## Introduction

Somatic cell nuclear transfer (SCNT) is one of the best methods available to reprogram differentiated cells so as to render them totipotent. The somatic nucleus is transferred into an enucleated oocyte that subsequently drives a deterministic reprogramming process [1], as opposed to the stochastic processes induced by reprogramming using either forced expression of defined factors, cell fusion, or nuclear incubation with cell extracts to induce pluripotency *in vitro* [2], [3], [4]. In all cases, cell fate reprogramming relies on the epigenetic remodelling of the initial chromatin architecture, either through DNA methylation, histone modifications, or nucleosome spacing, which leads to gene repression/de-repression and thus to molecular changes [5]. While all nuclear reprogramming techniques suffer from inefficiency, SCNT is the most efficient so far [2]. It is also the only method that addresses re-differentiation processes *in vivo* all the way through development to term. We examined re-differentiation processes prior to implantation using bovines because SCNT effectiveness is relatively high in this species [6] and their pre-implantation development period is long [7]. Indeed, although 20% to 60% of SCNT attempts in bovines result in blastocysts, the technique only results in live birth 1% to 10% of the time due to lethal defects and faulty reprogramming [8], [9], [10], [11], [12], [13], [14], [15]. Although SCNT blastocysts could perform equally well or better than controls in the weeks following embryo transfer at Day 7 [8], [16], elongation and gastrulation defects were reported prior to implantation (D19-D23). However, past studies have not examined more than one of these processes, elongation, gastrulation, or implantation, at a time. Indeed, no previous work has addressed elongation and gastrulation concomitantly [17], [18], [19], [20] and, even though SCNT conceptuses without embryonic discs have been found to elongate, as do trophoblastic vesicles [21], none have been subjected to molecular analyses [22].

We studied SCNT conceptus development prior to implantation to understand the embryonic/extra-embryonic interactions at work in differentiating blastocysts following their transfer to temporary recipient cows. In particular, we examined the elongation and gastrulation of Day 18 conceptuses. We based our descriptions of developmental patterns on previous observations of tissue differentiation and interactions from Day 12 to Day 25 that we gleaned from studies of artificial insemination (AI) or *in vitro* embryo production (IVP) [23], [24]. Based on these past results, we expected D18 extra-embryonic tissues should display a filamentous shape while D18 gastrulating tissues should harbour a primitive streak. In addition, extra-embryonic (EE) and embryonic (E) differentiations always appear to be synchronised [24], [25], [26], [27] and interdependent: i) the EE tissues pattern the E tissues and nourish them while interacting with the uterus [28], [29], ii) the E tissues contribute to EE elaboration via the differentiation of the epiblast into extra-embryonic endoderm and mesoderm [30], [31]. To evaluate these developmental characteristics following SCNT, SCNT conceptuses were compared to AI and IVP controls. Furthermore, to investigate the impact of reprogramming efficiency, three ear fibroblast donor cell lines (0029, 7711 and 5538) with similar levels of blastocyst *in vitro* production (at Day 7∶32.8%, 27.1% and 42.2%, respectively) but different calving success rates (Low: 1.8%, Med: 7.8% and High: 12.7%, [32], [33]) were selected. Overall, some SCNT conceptuses appeared normal while others showed atypical developmental features accompanied by a high incidence of embryonic/extra-embryonic discordance (or “uncoupling”). Elongation appeared to be secondarily affected as compared to gastrulation and was less associated with reprogramming efficiency at Day 21. The factors that define and restrict this efficiency and their relationship to genomic/epigenomic features at the somatic, embryonic, and extra-embryonic levels should be explored in the future.

## Results

To study the E/EE interactions at work in SCNT conceptuses beyond the blastocyst stage but prior to implantation, extra-embryonic and embryonic differentiation were individually analysed and correlated to pregnancy success rates at Day 21 and at term.

### Extra-Embryonic Differentiation

Conceptuses (n = 50) were placed in one of four elongation classes based on their length, width, and overall morphology. At Day 18, bovine conceptuses were expected to be 17–25 cm long, 1–2 cm wide at the centre, and showing signs of mesoderm formation at the latter location. If they complied with these criteria, they were classified as “filamentous”. If they were shorter in length (10–15 cm), they were “early filamentous”. Shorter and thinner conceptuses were “tubular” (<10 cm long, <1 cm wide) or “early tubular” (<3 cm long, <0.5 cm wide). Since the filamentous shape was the most frequent form observed at Day 18, filamentous conceptuses were considered “normal” (N1 and N2 classes, Table 1). Conversely, the tubular shape indicated a developmental delay, either mild (D class) or severe (Ab class). Severe elongation delays, as for conceptuses shorter than 3 cm, were considered “abnormal” at Day 18. When the elongation status of controls and SCNTs were compared, none of the conceptuses in the SCNT High, IVP, and AI groups displayed abnormal elongation, whereas those belonging to SCNT Med and Low groups did. This could have accounted for the lower implantation rates observed in these latter groups (51%, 57%) but its impact had to be assessed at the molecular level.

**Table 1:** Elongation classification and development success rates.

		Normal		Delayed	Abnormal		
		Filamentous (>15 cm)	Early Filamentous (10–15 cm)	Tubular (3–10 cm)	Early tubular (<3 cm)		
	N	N1	N2	D	Ab	**Implantation D21**	**Calving**
**AI**	10	10					
**IVP**	10	4	2	4		62%	55%
**SCNT High**	10	5	3	2		72%	13%
**SCNT Med**	10	3	2	2	3	51%	8%
**SCNT Low**	10	2	6	1	1	57%	2%

**Figure 1:**
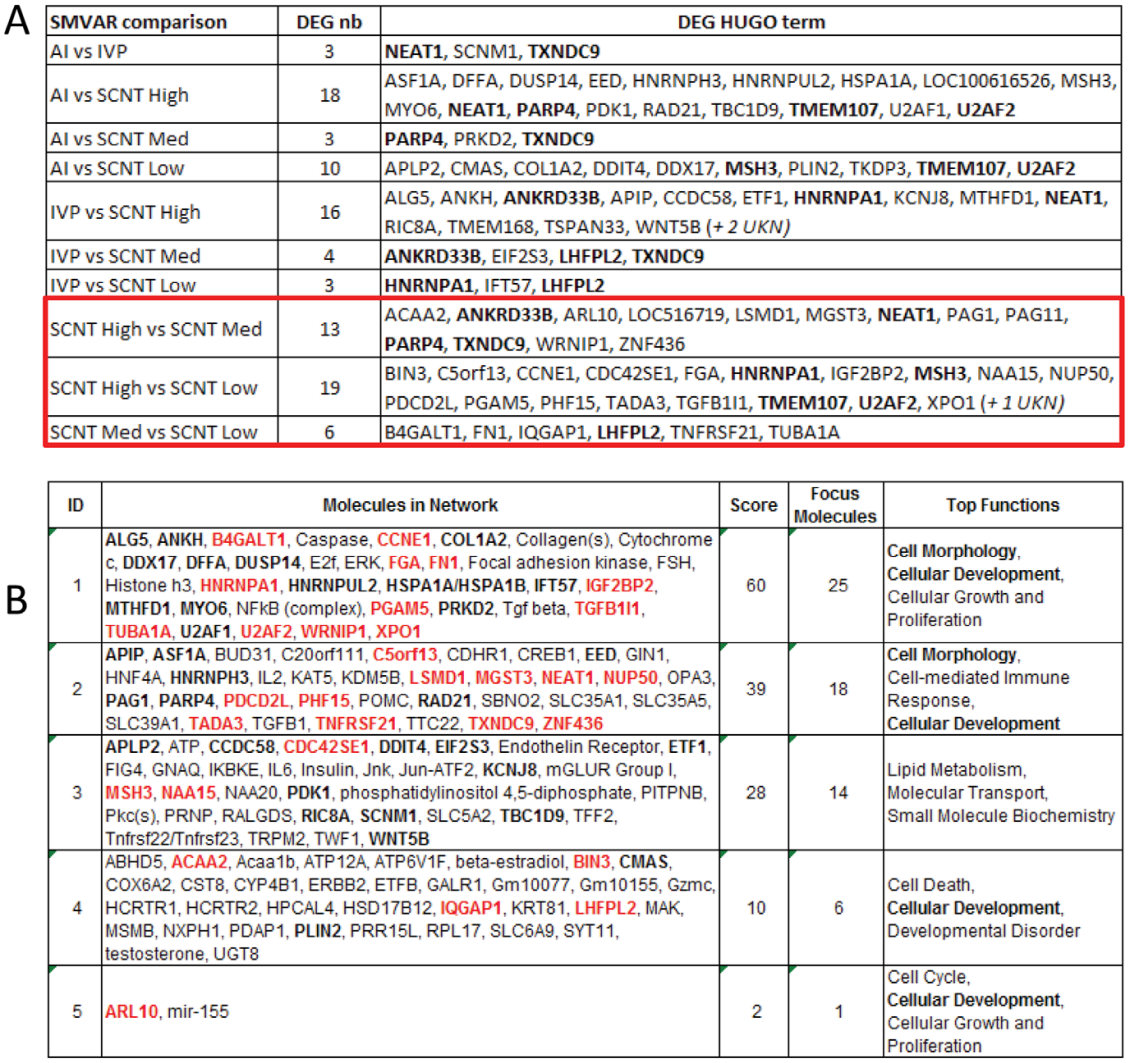
Differentially expressed genes (DEGs) among Day 18 EE tissues. A) Paired comparisons according to SMVar (Table S1). Across all the comparisons performed, 95 statistical occurrences were identified that corresponded to 72 unique DEGs. Multiple occurrences are in bold. Each gene ID is provided as a HUGO term. Among these DEGs, a few had been previously reported: single genes (*TUB1A1*, *B4GALT1)* or genes from the *CCDC, HSP or TKDP* families [17], [19]. B) IPA networks. The DEG list was analyzed with the Ingenuity Pathway Analysis software to identify the top gene networks and the pathways connecting them. SCNT-specific differences (in red) were found in three of four networks. In the IPA database, 4 proteins were located in the extra-cellular space, 8 at the plasma membrane, 20 in the cytoplasm, and 24 in the nucleus, of which 8 were recognised as transcription regulators.

Consequently, the molecular profile of each Day 18 conceptus (n = 50) was characterized while restricting the high throughput analysis to the extra-embryonic tissues. As each study group was represented by 7–10 elongated conceptuses, sampling was homogeneous enough to proceed to paired comparisons using the array data set. While the control groups were similar, the SCNT groups differed from each other, with the greatest number of gene expression differences occurring between the High and Low groups (19 DEGs, Fig. 1A). AIs, IVPs, and SCNTs had similar numbers of DEGs (18–16; 3–4), with the exception of SCNT Low (10–3). However, AI, IVP, and SCNT Low did not display higher intra-variability than SCNT Med or High (Table S1). As for individual genes, some were found to be pair-specific (*SCNM*1: AI-IVP pair) whereas others were shared by several pairs (*NEAT1* or *TXNDC9* for example). Nonetheless, all genes appeared to be connected through *in silico* networks and pathways (Fig. 1B), suggesting Day 18 SCNT-related alterations were the product of the same differentiation processes having been affected in different ways (especially cellular morphology and cellular development).

**Figure 2:**
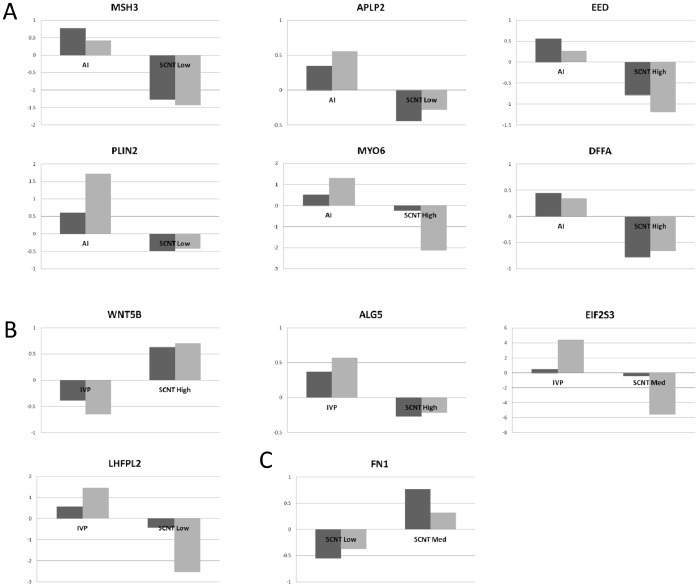
Validated gene expression differences. The gene expression differences are presented according to their decreasing rank (or adjusted P-value) within the SMVar output lists. In A: differences between SCNT and AI, in B: differences between SCNT and IVP, in C: differences among SCNT. The y-axis of the each histogram corresponds to the relative expression values of each DEG in EE tissues (AI, IVP and SCNT High, Med, Low). Array data are in grey, QPCR data in black.

**Figure 3:**
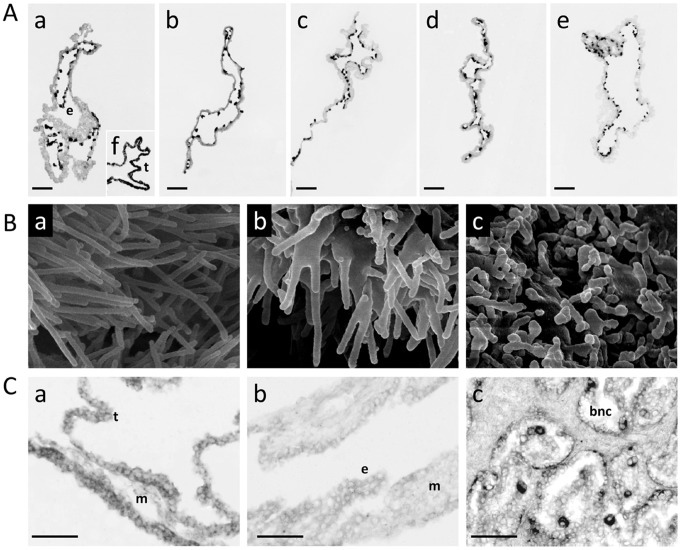
New biological outcomes of validated DEGs. A) *FN1* is restricted to the endoderm. *In situ* hybridisation in AI (a), IVP (b), and SCNT D18 EE tissues: Med (c), Low (d), High (e), using an anti-sense FN1 DIG-labelled riboprobe. (t) trophoblast, (e) endoderm. In the AI panel, f) shows c93/*SOLD1*, a trophoblast-specific control from [23]. The sense probe gave a negative signal in all tissues (not shown). Despite differential expression levels in array and QPCR data, this gene is expressed in the same cells, regardless of conceptus origin. Only a small part of each conceptus is shown. Scale bar is 150 µm. B) Microvilli abnormalities in SCNT EE tissues at D18. In the SCNT groups where *MYO6* and *LHFPL2* were underexpressed (b, c), epithelial microvilli appeared shorter and/or fused. SEM images of the external face (extra-embryonic ectoderm or trophoblast) of D18 EE tissues from SCNT Low and Med conceptuses as compared to controls (AI in a). Magnifications: a) x 30000, b) x 35000, c) x 30000. C) *PLIN2* is expressed in the trophoblast of D18 EE tissues and absent from the yolk sac at D25 [the yolk sac is composed of endoderm (e) and mesoderm (m)]. It is also expressed in binucleated cells (BNC) from D63 bovine placentas. BNC are differentiated trophoblast cells, often considered as the anatomical equivalent of mouse giant cells. *In situ* hybridisations with an anti-sense PLIN2 DIG-labelled riboprobe on tissue cross-sections from D18 EET (a), D25 Yolk sac (b) and D63 placentas (c) developed after AI. The sense probe gave a negative signal in all tissues; data are not shown. Only a small part of each tissue is shown. Scale bar is 100 µm.

Instead of focusing on a single gene network, we chose 4 networks (presented in Fig. 1B) and selected 14 genes based on their rank (or decreasing adjusted P-values) and their implication in: i) cellular morphology or development (*ALG5, APLP2*, *CCNE1*, *FN1*, *LHFPL2, MYO6*, *PLIN2, WNT5B*) and ii) alterations in chromatin modification or DNA repair (*ASF1A, BIN3, DFFA*, *EED*, *EIF2S3X, MSH3*). Validated expression differences are provided in Fig. 2; they mainly included differences between SCNT and control groups and within SCNTs (SCNT-AI: 6; SCNT-IVP: 4; SCNT Low-Med: 1). In addition, most of the differences resulted from weaker expression in SCNTs than in controls (8/9), except for *WNT5B*, which was more strongly expressed in SCNT High than in IVP. Non-validated DEGs were related to differences within SCNTs (Low-High: *BIN3*, *CCNE1*) or between SCNTs and controls (AI-SCNT Med: *LHFPL2*; AI-SCNT High: *MSH3*), except for *FN1*. To determine the cellular location of the validated transcripts in SCNTs as well as in controls, several *in situ* hybridisations were performed; three in particular revealed an interesting labelling pattern on the panel of tissue sections (*APLP2*, *FN1, PLIN2*). *APLP2* and *FN1* appeared to be restricted to endodermal cells and *PLIN2* to trophoblast cells (Fig. 3A, 3C). Using 2–3 conceptuses per group, we further determined that the cellular location of these genes was unchanged in SCNTs and IVPs (Fig. 3A), even in cases of delayed elongation (5 tubular shapes out of 9). Unfortunately, because the shortest conceptuses were extremely limited in size (<3 cm), gene cellular location could not be evaluated for abnormal elongations.

Three other validated DEGs were analysed because of their previously reported roles in the morphogenesis of epithelial microvilli (*MYO6, LHFLP2* [34], [35], [36]) or in the differentiation of mouse trophoblast giant cells (*PLIN2* [37]). We decided to analyse the microvilli of SCNT High (n = 4) and Low (n = 3) filamentous conceptuses given that the two groups more weakly expressed *MYO6* or *LHFPL2* and it is in the filamentous stage that microvilli have been described on trophoblast cells prior to implantation [29]. Nine filamentous conceptuses from the other groups (AI: 4, IVP: 2, SCNT Med: 3) were used as controls. Shortened and/or fused microvilli (Fig. 3B) were observed in some but not all SCNT High and SCNT Low conceptuses (2/4 and 1/3, respectively). Nothing similar was observed in any of the control conceptuses (n = 9). With regards to *PLIN2*, we confirmed that the transcript was confined to the trophoblast in AI samples and specifically to mononucleated cells at Day 18 and binucleated cells at Day 63. So as to indirectly evaluate the putative impact of gene expression differences at D18 on later extra-embryonic differentiation, the expression of other genes was analysed at D26, D36 and D63 in yolk sac and chorion tissue (n = 11, Fig. S1). The results sustained the initial *in silico* assessment: that cellular morphology may be affected at Day 18 and that SCNT-related alterations in gene expression levels at Day 18 may compromise cellular development in later stages. Nonetheless, further studies should test this functional hypothesis by employing gene invalidation in controls to mimic SCNT phenotypes.

In accordance with the elevated rate of occurrence of elongated conceptuses in SCNT and controls (46/50), very few molecular differences appeared significant (when comparing to the number of genes expressed in Day 18 extra-embryonic tissues: 72/9162). To evaluate embryonic differentiation and compare gastrulation success rates in SCNTs and controls, we did not search for DEGs in embryonic tissues at Day 18 but rather assessed gastrulation more globally with a staging method that has been used in chicks, rabbits, pigs, and cows [24].

### Embryonic Differentiation

**Figure 4:**
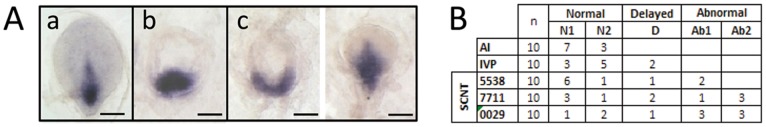
Gastrulation patterns. A) Definition of gastrulation classes. Normal *Brachyury* patterns are shown in N1 (a) and D (b) embryonic discs. Abnormal *Brachyury* patterns (U-shaped and broadened labelling) are shown in Ab1 (c) embryonic discs. These are whole-mount *in situ* hybridisations with an anti-sense Brachyury DIG-labelled riboprobe performed on embryonic discs from two SCNT High (a, b) and two SCNT Low conceptuses (c, right and left panels). Scale bar: 100 µm. B) Overview of all conceptuses.

Relying on morphological signs of gastrulation and the expression pattern of an early mesoderm marker, the *Brachyury* gene, this staging method describes D18 patterns in AI conceptuses as being at stage 4 or 3 (referred to as normal: N1 and N2, respectively; Fig. 4). Accordingly, these stages were the most frequently observed and their presence best predicted successful development to term in AI, IVP and SCNT High groups (Table 1B). When fewer N1 and N2 stages were observed, more unusual patterns were recorded including late and abnormal development. In SCNT Med and Low conceptuses, a few embryonic discs were delayed but normal, i.e. they appeared morphologically younger than those in controls (AI), and corresponded to stage 2 [24]. They were thus recorded as delayed (D). Since the D pattern occurred with equal frequency in all groups except for AI, this delay in embryonic development was not typical of post-SCNT differentiation. Conversely, several other discs from SCNT Med and Low groups were atypical, i.e. not previously observed in controls [23], [24], [31], [38]. Depending on the severity of their phenotype, they were called Ab1 or Ab2. In the mild phenotype (Ab1), discs were small or folded, with atypical U-shaped or broadened *Brachyury* labelling (Fig. 4A). In contrast, the severe phenotype (Ab2) displayed no disc, or at least none that was morphologically recognisable or molecularly detectable using the *Pou5f1* gene as a molecular marker for the epiblast [23]. The highest numbers of Ab1 and Ab2 patterns were found in those conceptuses that had the lowest success rates at Day 21 and at term, namely those in the SCNT Med and Low groups (Table 1).

Our observations of embryonic tissues showed that post-SCNT differentiation partly affected embryonic functions. However, the likelihood of normal gastrulation was lower than that of normal elongation (38/50 *versus* 46/50). We thus compiled all our classifications of elongation and gastrulation and counted cases of co-occurrence (30 out of 50), where the extra-embryonic and embryonic tissues were both recorded as normal, delayed or abnormal (NN, DD, AbAb; Table 2). These cases included all AIs (10/10), most IVPs (6/10) and SCNT Meds (7/10), but only a few SCNT Highs (4/10) and Lows (3/10). The other cases where extra-embryonic and embryonic tissues were in discordance (n = 20) included the 12 cases initially observed (normal elongation with delayed, abnormal, or very abnormal gastrulation) but also 8 new cases where gastrulation appeared normal but was associated with delayed or abnormal elongation. Out of these, 6 came from the SCNT groups [High: 2, Med: 3, Low: 1] and 2 from the IVP group. In general, few discordances were mild (normal versus delayed or ND, n = 9) and most were severe (normal versus abnormal or NAb, n = 10). Severe discordances were only found in the SCNT groups (High: 3, Med: 3, Low: 4) and were correlated with a higher probability of embryo loss at implantation for the High and Low groups (28%, 43%; Table 1).

**Table 2:** Uncoupling during elongation and gastrulation.

	EE morphology				
	Normal		Delayed	Abnormal	
	Fil (>15 cm)	Early Fil (10–15 cm)	Tub (3–10 cm)	Early tub (<3 cm)	
**AI**	N1 (5), N2 (5)				**E** **morphology**
**IVP**	N1 (2), *D(2)*	N1 (1), N2(1)	*N1 (2)*, D (2)		
**SCNT High**	N1 (2), N2 (1), **Ab1 (2)**	N1 (1), *D (1)*, **Ab1 (1)**	*N1 (2)*		
**SCNT Med**	N1 (2), **Ab1 (1)**	N1 (1), **Ab2 (1**)	D (2)	**N2 (1)**, Ab2 (2)	
**SCNT Low**	N1 (1), *D (1)*	N1 (1), *D (1)*, **Ab1 (1)**, **Ab2 (3)**	**Ab1 (1)**	Ab2 (1)	

Mild uncoupling events are in italics, severe uncoupling events are in bold, and coordinated E/EE differentiations are in normal font. Coordinated E/EE differentiations include normal/normal, delayed/delayed, and abnormal/abnormal morphologies.

### Embryonic/extra-embryonic Discordances

**Figure 5:**
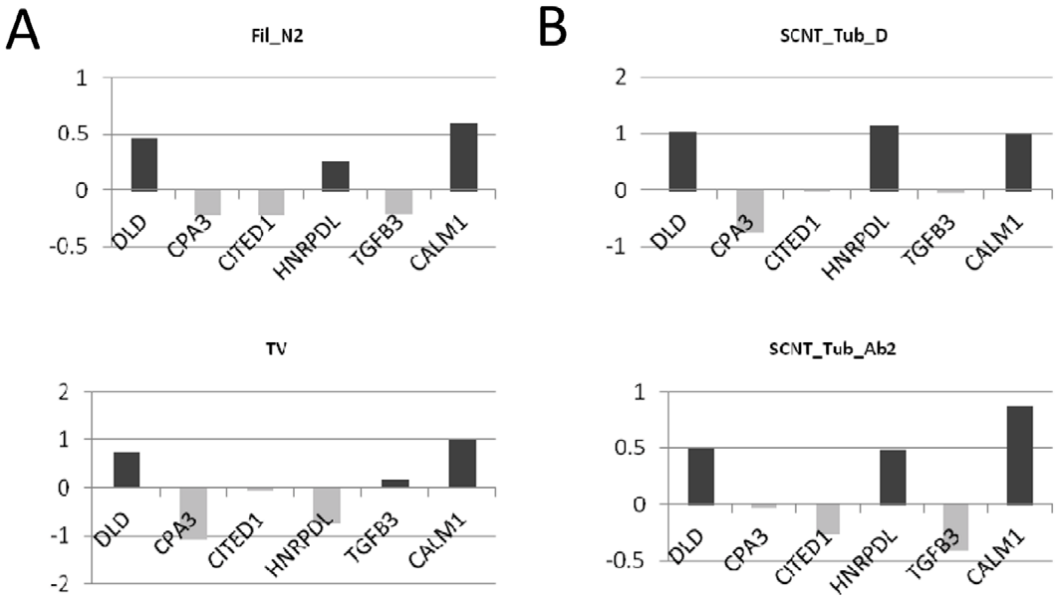
Molecular uncoupling of E and EE tissues in SCNT conceptuses. The gene set used to classify each conceptus includes six genes from the extra-embryonic tissues and has been described as an accurate predictor of embryonic stages in controls. A) Patterns resulting from AI: examples of a filamentous conceptus with a disc at stage 3 (Fil-N2) and a trophoblastic vesicle (TV) with an ablated disc since Day 15. B) Patterns resulting from SCNT: examples of tubular conceptuses with a delayed embryonic disc (stage 2) or no disc (Ab2).

To build on these discordance data obtained from macroscopic observations, we used a small extra-embryonic gene set that was previously identified as an accurate predictor of the embryonic stages in controls at gastrulation (AI [24]). When elongation and gastrulation were delayed, the extra-embryonic gene set displayed similar patterns in the SCNT and AI groups despite them being in different embryonic stages (Tub-D *versus* Fil-N2 phenotypes; Fig. 5). In addition, with or without embryonic tissues, tubular SCNT conceptuses were similar. There was thus a discrepancy between the molecular and morphological results, which highlighted another important difference. Despite having a morphology similar to trophoblastic vesicles (TV), SCNTs without discs did not display the extra-embryonic pattern of TVs, but that of tubular SCNTs, irrespective of their embryonic stage (delayed or normal).

**Figure 6:**
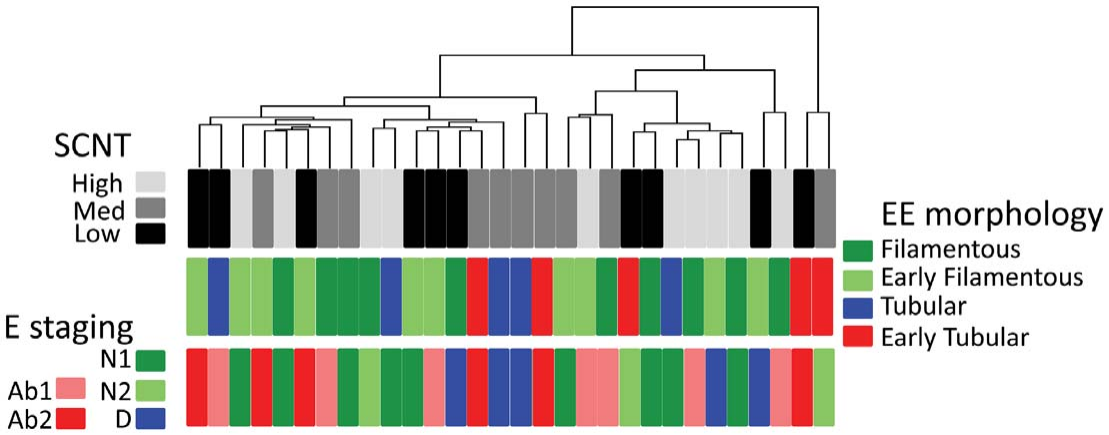
Molecular and morphological uncoupling of E and EE tissues in SCNT conceptuses. A hierarchical clustering was first performed using the 500 most variant genes from SCNT High, Med, and Low groups. The 30 SCNT conceptuses are presented with their EE and E morphology. Cases of coupled E/EE differentiations were rare. See complementary details in Table 2.

To add to these molecular results, a hierarchical clustering was performed on the 500 most variant genes expressed by SCNT extra-embryonic tissues (n = 30). When the results were analysed in relation to EE morphology and E staging (Fig. 6), discordant patterns were apparent (same numbers, same groups). However, these discordances were not easily linked with two new factors: i) the sex of the conceptus (all SCNTs are females): the absence of a sex effect was confirmed by statistical analyses on AIs (males versus females) as well as on females only (AI-IVP-SCNT; Table S1) and ii) the reprogramming efficiency: SCNT High and Med groups both displayed the lowest number of severe E/EE uncoupling events (3/10) but not the same level of implantation failure (28% and 49%, respectively).

### Abnormal SCNTs

**Figure 7:**
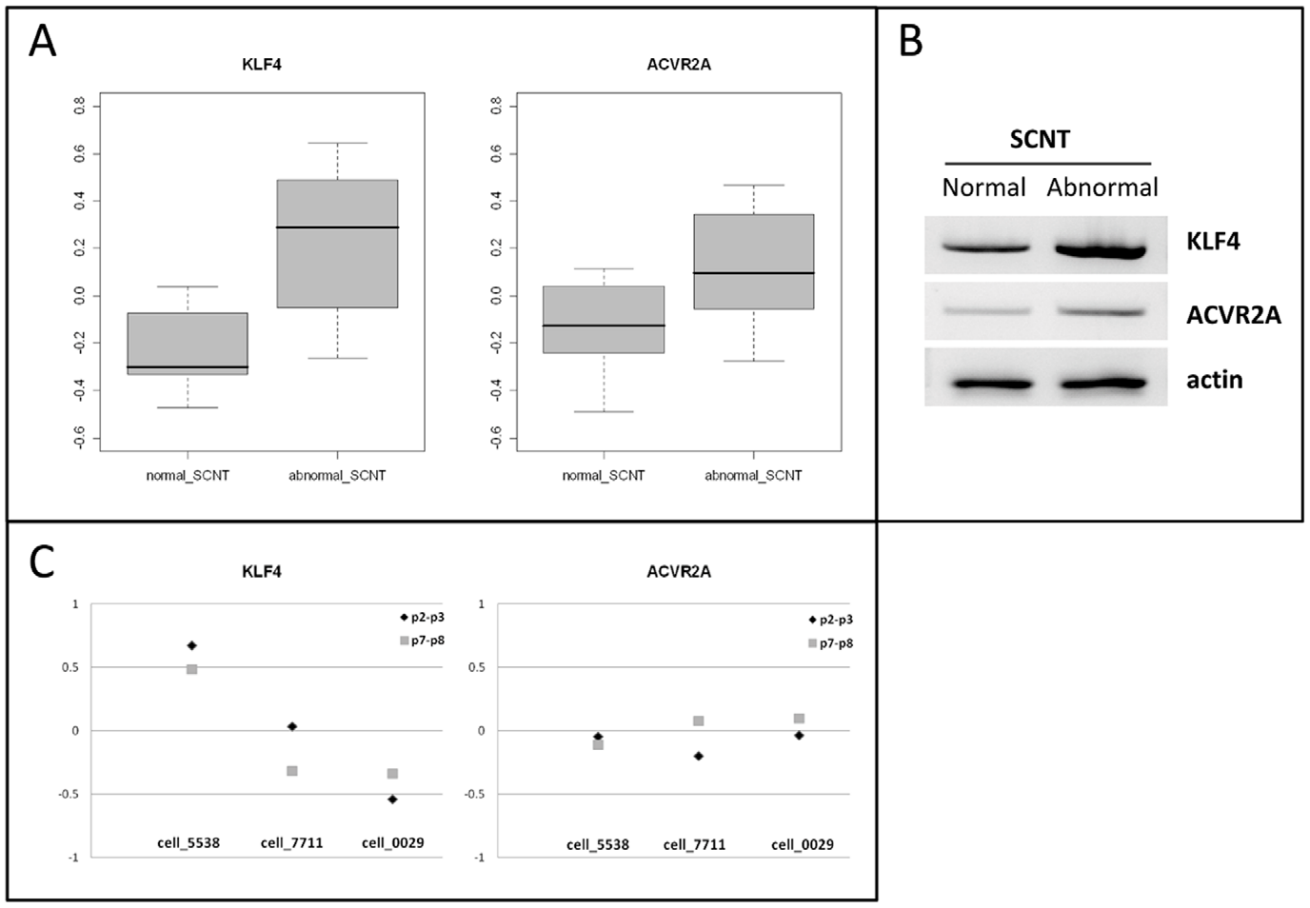
*KLF4* and *ACVR2A* discriminate among normal and abnormal SCNTs. A) Box plots of *KLF4* and *ACVR2A* expression in normal and abnormal SCNTs. B) PCR results from normal and abnormal EE tissues, pooled as indicated in Table S1. C) Expression of *KLF4* and *ACVR2A* in the fibroblasts (5538, 7711 and 0029) used to generate SCNT High, Med, and Low conceptuses. Triangles correspond to cell passages temporally proximate to biopsy (p2-p3), squares to the passages closer to nuclear transfer (p7–p8).

Over the totality of the SCNT conceptuses, half were normal and half were abnormal. To be able to discriminate between them and preserve embryonic tissues for other analyses, we searched for extra-embryonic genes that could distinguish between normal and abnormal SCNTs. Using three classification methods and taking into account the genes commonly identified by these methods, two genes were identified that could serve this function: *KLF4* and *ACVR2A* (Table S1; Fig. 7A). Both were more strongly expressed in abnormal versus normal SCNT extra-embryonic tissues. Because the SCNT High, Med and Low groups all contained normal and abnormal conceptuses, differences in expression of these two genes were unlikely to stem from differences in somatic origin. Instead, they could have indicated poor post-SCNT differentiation. Moreover, these genes displayed different expression patterns in somatic cells (Fig. 7C); *KLF4* was highly expressed in the 5538 fibroblasts (as compared to the others: 7711, 0029). It thus seemed that *KLF4* was downregulated in normal Day 18 conceptuses from the SCNT High group but upregulated in abnormal conceptuses from SCNT Med or Low groups (as compared to its initial levels in the corresponding fibroblasts). Conversely, *ACVR2A* seemed upregulated in abnormal conceptuses (as compared to its level in different somatic cells; Fig. 7A, 7C).

## Discussion

In the current study, some SCNT conceptuses appeared normal while others demonstrated features signalling atypical elongation or gastrulation. Some of these features correspond to those previously reported in studies examining only one of the two developmental processes, either elongation or gastrulation [17], [18], [19], [20], [22], [39], [40], [41]. Here, however, we describe for the first time a high incidence of E/EE uncoupling, a result which emerged from our concomitant analysis of the two processes. Our use of somatic cell lines with different reprogramming efficiencies (High, Med, or Low) allowed us to ask questions about the putative links between SCNT-related uncoupling and embryo loss and contrasts with most studies’ use of a single cell line (often comparable to SCNT Med).

### Uncoupling Compromises Development

The uncoupling of embryonic and extra-embryonic differentiation was not only apparent in SCNT conceptuses with no disc but also in all abnormal conceptuses whatever their somatic origin. Severe uncoupling was found in all SCNT groups and was correlated with a high probability of embryo loss at implantation for the High and Low groups. Similarly, mild uncoupling was observed in all SCNT groups and in the IVP group (4/10). Whether mild uncoupling may account for embryo loss at implantation in IVPs (40%) is unknown. However, an asynchrony of more than 36 h to 48 h between the development of the conceptus and that of the uterus has long been reported as detrimental when the uterus is ahead of the blastocyst at the time of transfer [42], [43] but beneficial when the uterus lags behind [44]. The state of the uterus is thus another factor to consider when predicting implantation success due to its sensor/driver capacity [45], [46]. To evaluate a putative “maternal” effect on the conceptuses that we studied, two statistical analyses were performed (Table S1) that evaluated the effect of a) being developed in a recipient cow and b) the specific breed of the recipient. Neither effect was significant. Moreover, as illustrated by SCNT Med group, in which the embryo loss at implantation (43%) exceeded severe uncoupling (3/10), pre-implantation loss could originate from mild uncoupling. Nonetheless, the SCNT conceptuses from medium and low efficiency groups had the highest occurrence of abnormal embryonic tissues and the lowest pregnancy success rates at Day 21 and at term.

### Embryonic Tissues Show Diverse Effects

Among the embryonic defects observed, two major classes appeared: one affecting the primitive streak (its shape or position within the disc; Ab1 class) and the other the disc itself (Ab2 class). In the first class, where a streak appeared along with the nascent mesoderm, an epiblast was formed and proper antero-posterior patterning took place. Gastrulation was thus not totally impaired but rather displayed atypical patterns. Unusual, similarly shaped, or broadened *Brachyury* labelling patterns were reported and interpreted as defective specifications of the primitive streak due to defective epiblast cells or defective hypoblast signals. Indeed, signals emanating from the hypoblast not only normally position the streak but also restrict its size and enlargement [47], [48], [49]. The Ab1 class could thus originate from post-SCNT defects affecting the hypoblast or its signalling capabilities. The hypoblast is defined in the current work as it was originally in the chick system: it is comprised of the cells underlying the epiblast in the embryonic disc. In the second class, where no embryonic disc was found, the mechanism underlying the defect likely occurred prior to gastrulation and affected the epiblast lineage (due to defective proliferation or pluripotency), the E/EE ratio (as described in some classes of mouse SCNTs where the EE tissues overgrew [50]), the ICM lineage (due to defective proliferation or pluripotency), or the ICM/TE ratio at the blastocyst stage. As no extra-embryonic overgrowth was observed in SCNT conceptuses without discs, the Ab2 class may originate from post-SCNT alterations in the ICM/epiblast lineage or the ICM/TE ratio. Based on past and present work, two lines of reasoning favour this hypothesis. The first is the lack of molecular similarity between Trophoblastic Vesicles (TV) and SCNTs without discs. Ab2 conceptuses may lack a healthy disc for a longer period of time than TVs (experimental ablation of the disc at D15); alternatively, they may never form a healthy disc or only transiently so. The second is the high percentage of conceptuses without discs at Day 14 post-SCNT [22]. To further support this hypothesis, producing SCNTs at Day 14 (or Day 7) to characterise their epiblast (or ICM) would be useful. So far, although no Day 14 SCNT epiblast has been molecularly characterised, Day 7 blastocysts have been. These analyses did not reveal altered gene expression in SCNT groups (High, Med or Low) relative to controls (AI or IVP) that could be linked to ICM pluripotency issues, ICM/TE ratio, or early cell fate decisions [51]. However, ICM cells were not specifically scrutinised. The derivation of embryonic stem cells from SCNT High, Med, or Low groups at Day 7 or Day 14 could also provide functional clarification. While this is not yet feasible in cattle [52], it has been successful in pigs [53] and mice (EpiSC from controls and NT embryos [54]).

### Extra-embryonic Tissues Seem Secondarily Affected at Day 18

In contrast to the extensive embryonic defects observed in SCNTs and controls, their EE tissues were similar, suggesting that they are less affected by the SCNT process and that their defects contributed less to pregnancy loss at Day 21. Indeed, with their high rate of successful elongation and few DEGs, Day 18 EE tissues looked normal regardless of their somatic origin. Other reports described similar results for other somatic backgrounds at Day 15 or 17: most genes showed equal expression levels between SCNTs and AIs [55], [56] or SCNTs and IVPs [40], [57], [58] such that small DEG ratios were noticed: 18/1206 [17] or 47/1321 [19]. However, the DEGs we found could affect the absorption of nutrients from the uterine histotroph [59] via altered microvilli or could change the interactions within extra-embryonic cell layers (differentiation, motility or adhesion [60]) by altering the extra-cellular matrix. As hypothesised for the embryonic tissues (where disc absence at Day 18 may originate in earlier defects), it may be that Day 18 extra-embryonic defects become more visible at implantation due to trophoblast-uterus contacts (as in the mouse [61], [62]). Reports that describe placental defects and link them to defective trophoblast development also support this possibility [63]. Nonetheless, to clarify the role of each cell layer (trophoblast, endoderm or mesoderm) in generating SCNT abnormalities affecting the yolk sac or the chorion, there will be a need for: i) extended localisation of SCNT-affected transcripts and ii) in-depth transcriptome analyses via micro-dissection or RNA sequencing.

### Somatic Origins, Reprogramming, and Re-differentiation Matter

**Figure 8:**
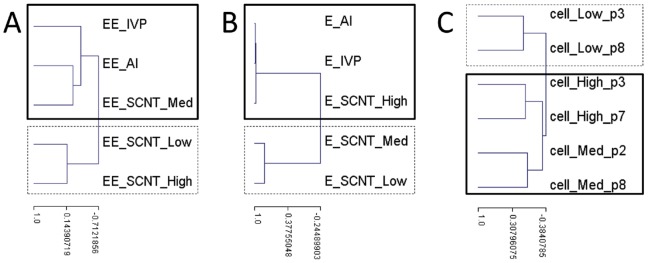
Differential clustering of differentiating tissues and somatic cells. Ranking of SCNTs and controls based on the EE profiles (A) or the E stages (B). Ranking of the fibroblasts (5538, 7711 and 0029) based on their molecular profiles and using the 500 most variant genes among them. The cell passages (p) more temporally proximate to biopsy (p2 to p3) versus nuclear transfer (p7 to 8) were compared. Each ranking series is represented by a dendrogram.

Despite the similarity in AI, IVP, and SCNT High gastrulation patterns, the latter group nonetheless demonstrated relatively different gene expression in elongating tissues. These differences may reflect quicker cascades of transcriptional regulation or simpler epigenetic architectures that result in SCNT High conceptuses having greater EE plasticity and higher implantation success rates than IVPs (at D21). Given the stronger expression of most DEGs and the weaker expression of *EED* (which encodes a protein of the Polycomb repressive machinery [64]) in the SCNT High group, higher molecular plasticity is plausible. In contrast, SCNT Med and Low groups differed less from controls in their molecular profiles yet evidenced abnormal embryonic patterns. As a result, when SCNT groups were ranked according to their similarity to controls, a different order was obtained when E versus EE differentiation was used. Furthermore, none of the rankings matched the one obtained using the somatic cells (Fig. 8). As the difference between cell passages was less important for a given cell than the difference between cell donors, our guess is that *in vitro* culturing differences were of less consequence than cell line characteristics.

The differential performance of the different cell lines with regards to both SCNT reprogramming and *in vivo* development up to implantation support a distinction in their reprogramming capabilities. Nevertheless, each somatic cell line gave rise to abnormal conceptuses at Day 18 that shared an increased expression of *KLF4* and *ACVR2A* that was independent of their expression in the fibroblasts (Fig. 8). Although *ACVR2A* plays a major role in human trophoblast differentiation and has been linked to preeclampsia susceptibility in humans [65], [66], [67], it is unclear how it could contribute to abnormal SCNT phenotypes. On the other hand, *KLF4* encodes a transcription factor that is essential for inducing pluripotency in stem cells (or iPS; when overexpressed with other factors [68], [69]) and maintaining cancer stem cells [70]. In cultured cells, its expression has also been temporally associated with conditions that promoted growth arrest, such as serum deprivation [71]. Consistent with this finding, the 5538 fibroblasts were those that responded well to serum deprivation (93% switching to G0/G1) and expressed greater amounts of *KLF4*. Alternatively, this pattern could relate to transcriptional regulation and/or epigenetic marks since *KLF4* is upregulated by HDAC inhibitors in cardiomyocytes [72] and in abnormal Day 18 SCNTs but downregulated in normal ones.

Deciphering the contribution of somatic origins versus reprogramming errors to the generation of defects is the next challenge. Although abnormal Day 18 phenotypes could be due to faulty reprogramming, the molecular analyses of SCNT High, Med and Low blastocysts revealed that proper somatic-to-embryonic reprogramming had occurred [32], [51]. At earlier developmental stages, however, abnormal molecular patterns were detected in morulae from abnormal SCNT Low conceptuses [73]. Abnormal Day 18 phenotypes may thus appear during post-blastocyst differentiation. E and EE functioning would thus be altered and/or uncoupled, compromising development to term. The contrasting phenotypes observed in the SCNT Low and High groups raise questions about the epigenetic status of E/EE tissues and bovine fibroblasts. Indeed, recent work in mice highlights the importance of early epigenetic marks linked to epiblast size, gastrulation features, and E/EE relationships. They also showed that modifying these factors could rescue compromised developments and increase reprogramming efficiency [74], [75], [76].

## Materials and Methods

### Sample Collection

Animal care and procedures were completed in accordance with EU directives and the authorization of the French Ministry of Agriculture (B91332). The protocol is registered as protocol 06-002 and was approved by the Regional Ethical Committee of Paris-Sud. The authorizations allowing *in vitro* embryo production and embryo transfer were delivered by French Veterinary Services (N°FRPB780 and FRTB910).

#### Animals

The cattle used in the present experiment were held at the experimental farm of INRA Bressonvilliers. The donors of the SCNT cell lines were 3 Holstein females. The recipients of SCNT (n = 70) or IVP embryos (n = 16) as well as the females bred by AI (n = 14) were dairy or crossbred cows proven to be fertile (at least one calving event prior to the experiment).

#### Somatic cell nuclear transfer and In vitro production

Primary cultures of adult bovine fibroblasts were established from ear skin biopsies of 3 Holstein heifers (0029, 7711 and 5538 [77]). The cell lines that were used for SCNT were derived from the primary cultures and came from passages 6 to 12. Donor fibroblasts were grown to confluence and synchronised to G0/G1 of the cell cycle before nuclear transfer. Recipient oocytes were matured *in vitro* and enucleated at 20–22 hrs post-maturation (hpm). Reconstructed embryos were fused by electrostimulation and activated in 10 µg/ml cycloheximide and 5 µg/ml cytochalasin B for 5 hrs after fusion, then co-cultured on Vero cells for 7 days at 39°C under 5% CO_2_ in micro-drops of B2 medium supplemented with 2.5% FCS.

Control IVP embryos were obtained from the same batches of *in vitro* matured oocytes. Twenty-four hours after the onset of maturation, metaphase II oocytes were incubated with heparin-capacitated, thawed spermatozoa in TALP medium for 18 hr according to the standard *in vitro* fertilization technique used in the laboratory. After IVP, the embryos were cultured until Day 7 under the same conditions as nuclear-transferred embryos.

#### Embryo transfer

All embryo transfers were performed in the same facility by the same specialists (CR and YH) into the uterine horn ipsi-lateral to the corpus luteum. Due to the low probability of successful implantation by SCNT Low conceptuses, 5 or 6 SCNT blastocysts were transferred per recipient in order to recover at least one conceptus by Day 18. Although the SCNT High and IVP groups performed better, we nonetheless used the same protocol for all groups: 5 or 6 IVP or SCNT Day 7 blastocysts (all of grade 1 or at least 4 grade 1+1 or 2 grade 2).

#### AI controls

In order to get *in vivo* control conceptuses, a group of 14 cows were bred by AI after induced oestrus. Synchronisation treatment included a mild superovulation treatment (600 UI PMSG) so that control females better corresponded to the recipient females that received 5 to 6 blastocysts. AI was performed using the same batch of frozen sperm from a single Holstein bull as for *in vitro* fertilization and IVP embryos.

#### Embryo collection

On Day 18 of gestation, AI, IVP, and SCNT conceptuses were non-surgically collected using a modified IMV catheter and by gently flushing the uterus with warm PBS. After flushing, each recovered conceptus was carefully rinsed in fresh PBS before treatment. For each conceptus (n = 50, 10 per group), the embryonic disc was dissected and fixed in 4% paraformaldehyde whereas the extra-embryonic tissues were either fixed or snap-frozen in liquid nitrogen before storage at -80°C until RNA extraction.

#### Additional samples

On Days 25, 36, and 63 of gestation, AI samples including the yolk sac, chorion and placenta were collected immediately after slaughter and fixed in 4% paraformaldehyde. *In situ* hybridisation was then performed on each of these tissues and samples obtained from at least two pregnancies.

### Embryo Sexing

Genomic DNA was obtained from each AI and IVP conceptus after total RNA extraction from extra-embryonic tissues using Trizol (as per the manufacturer’s instructions). The sex of the embryos was genetically determined by PCR using primers R-IV/U-IV and BTANRP1/2 as described in [78]: R-RIV 5′-GTT TTA TTA TCC CAG CAAG-3′, U-IV 5′-TAT TCC TTT GGG GAG CA-3′, BTANRP1 5′-CCA ACT TTC CCT TCT TTC CC-3′ and BTANRP2 5′-ATG GCC CAA AAG AAC ATT CA-3′. Briefly, primers R-RIV/U-IV amplified a 655bp Y-specific sequence present in male genomic DNA and primers BTANRP1/2 amplified a 370pb BTANRP sequence present in all tested samples, thus showing the effectiveness of the PCR reactions. DNA was amplified using an initial denaturising step at 94°C for 5 min, followed by 35 cycles of denaturation at 94°C for 45 s, annealing at 55°C for 45 s, and synthesis at 72°C for 45 s. An extension time of 5 min was added at the end of the final cycle.

### RNA Extraction and T7 Linear Amplification

Total RNA from AI, IVP, and SCNT EET (n = 50) was isolated using Trizol (Invitrogen). Linear amplification was performed using MessageAmp aRNA kit (Ambion, Courtaboeuf, France), starting from 1 µg total RNA as in [79]. Two passages of the 5538, 7711 and 0029 fibroblasts were similarly treated to extract and amplify RNA.

### Bovine 10 K Array

This array (GPL7417) was partly developed in the laboratory. It contains 7800 cDNA inserts from term placenta [80] and 2400 cDNA inserts from extra-embryonic tissues of bovine embryos (D14–D24) as well as young foetuses (D36 and D64). The corresponding libraries are indexed in Unigene as Lib. 3743, 17188, 15992. Internal controls (n = 30) were also included in the array. Finally, 10,214 unique cDNA were spotted onto Nylon NC membranes (AmershamBiosciences) with a 3×3 pattern (QBot; Proteigene, Saint Marcel, France) at the CRB GADIE (INRA, Jouy-en-Josas).

### Array Hybridisation, Image Acquisition, and Quantification

Array hybridisation was as described in [79]. Briefly, 500 ng of amplified RNA (aRNA) were labelled with [a33P]dATP by RT and hybridised to each membrane. Membranes were then exposed to phosphorscreens for 7 days. The hybridisation signals were quantified with the Imagene 5.5 software (BioDiscovery, Proteigene) on the ICE platform (INRA, Jouy-en-Josas). The hybridisation with the extra-embryonic tissues gave rise to the GSE34944 data set in the Gene Omnibus database (http://www.ncbi.nlm.nih.gov/geo/). The hybridisations with the 5538, 7711 and 0029 fibroblasts (two passages each, about a million cells) gave rise to a data set with only one biological replicate and thus were not deposited in the GEO database.

### Gene Expression Data Analysis

The GSE34944 including the raw data on Day 18 EET from the AI, IVP, and SCNT groups was mean-centred and log-transformed. The differential analyses performed on this data set were done using a structural mixed model for variances (SMVar [81]) with different False Discovery Rates (FDR), as indicated in Table S1. Differential expressions were then ranked according to their P-value corrected for multiple testing (due to the thousands of genes per array; Benjamini-Hochberg adjustment), or adjusted P-value. Other paired comparisons were performed as needed to assess intragroup variability, sex effects, recipient effects, or embryonic stage effects (Table S1). A discriminative analysis was also performed on normal and abnormal SCNTs using different classification methods (RF, CART and SVM as reported in [24]). Supervised hierarchical clustering was also used and employed Euclidean distance and complete linkage (TMev 4.7.3).

### Real Time PCR

Real-time PCR was carried out in a final volume of 15 µl with 1 µl of diluted reverse transcriptions in a 1x SYBR green Master Mix (Applied Biosystems) with 0.3 µM of gene-specific primers (Table S2). Reactions (n = 25, 5 per group) were run in duplicate using an ABI Prism 7000HT (Applied Biosystems). The presence of a specific and unique PCR product was checked with ABI Prism melting curves. For normalization, GAPDH, β-actin, RPL19, and RPS18 were used as endogenous controls [82]. Relative quantification was calculated using qBasePlus Software (Biogazelle).

### *In situ* Hybridization

#### Whole-mount *in situ* hybridization

The isolated embryonic discs that were dissected from AI (n = 10), IVP (n = 10), or SCNT (n = 3×10) conceptuses were fixed in 4% paraformaldehyde, stored, and hybridised with a Brachyury-DIG-labelled riboprobe as in [38]. The hybridized embryos were mounted in mowiol and photographed with a Coolsnap camera (Photometrics). The embryo staging was as in [24].

#### *In situ* hybridization on tissue sections

Extra-embryonic tissue sections (10 µm) from AI (n = 17), IVP (n = 4), and SCNT (n = 9) conceptuses at Day 18 were hybridized as described in [23], using DIG-labelled riboprobes (RNA DIG-labelling kit, Roche Diagnostic). Sense and antisense riboprobes were also prepared as in [24], using specific primers adapted to i) the plasmid of each cDNA library to generate a cDNA template prior to *in vitro* transcription and ii) the orientation of each cDNA to synthesise the proper sense and antisense probes (Table S3). Of the ESTs of interest that were initially annotated (n = 45), 4 gave either no DNA template or no riboprobe, 5 gave similar results with the sense and antisense probes, 2 gave distinct signals with the sense and anti-sense probes, 18 gave weak signals, 4 gave fuzzy signals and were thus left over. Finally, 22 cDNA fragments that were i) isolated from the 10 K cDNA libraries, ii) successfully transcribed *in vitro*, and iii) provided clear sense/antisense signals, were considered. Among them, 8 were ubiquitous whereas 14 displayed restricted cellular locations. Two controls were used: the *c93*/*SSLP1 or SOLD1* [23], [83], [84] and *Pou5f1/Oct-4* cDNA [23] (JF919673 and DQ126156 accession numbers, respectively). Slides were scanned with a Nanozoomer (Hamatsu, France) and exported as.tif images.

### Scanning Electron Microscopy

A piece of each EE tissue was rinsed with phosphate-buffered saline (PBS) and fixed in 2.5% glutaraldehyde (in 0.1 mol/l cacodylate buffer, pH 7.4) for 30 min at room temperature. After having been washed repeatedly with distilled water, samples were dehydrated in an ascending series of ethanol and dried at the critical point using carbon dioxide as the transitional fluid. Dried samples were then coated with a conductive layer of carbon. Observations and photographs were made using a Hitachi S4500 scanning electron microscope set at 8 or 10 kV.

## Supporting Information

Figure S1 **Additional ISH on AI samples from Days 25 to 63**. A) The yolk sac at D25 (a) or D36 (b, c) is composed of endoderm (e) and mesoderm (m). For example, *FN1, FGA, U2AF2, DUSP14, ANKRD33* were expressed in the endoderm at these stages. Here, only *FN*1 is illustrated (a, b). Other genes like *B4GALT1, PAG11 or TKDP3* were absent from the yolk sac (as in c). B) The chorion at D63 (a-c) is composed of trophoblast (t) and mesoderm (m). Most of the genes tested were expressed in the trophoblast as in (a), namely *B4GALT1, DFFA, PAG11, TADA3, TUB1A1.* Only a few showed a different pattern, as illustrated by *TKDP3* in the trophoblast (b) or *COL1A2* in the mesoderm (c). Another example is provided by *PLIN2* in Fig. 3.
(TIF)

Table S1 **Statistical analyses.**
(DOC)

Table S2 **Primers for QPCR.**
(DOC)

Table S3 **Primers for DNA templates before** ***in vitro*** **transcription.**
(DOC)

## References

[1] Jullien J, Pasque V, Halley-Stott RP, Miyamoto K, Gurdon JB (2011). Mechanisms of nuclear reprogramming by eggs and oocytes: a deterministic process?. Nat Rev Mol Cell Biol.

[2] Hanna JH, Saha K, Jaenisch R (2010). Pluripotency and cellular reprogramming: facts, hypotheses, unresolved issues.. Cell.

[3] Hochedlinger K, Plath K (2009). Epigenetic reprogramming and induced pluripotency.. Development.

[4] Maruotti J, Jouneau A, Renard JP (2010). Faithful reprogramming to pluripotency in mammals - what does nuclear transfer teach us?. Int J Dev Biol.

[5] Gaspar-Maia A, Alajem A, Meshorer E, Ramalho-Santos M (2011). Open chromatin in pluripotency and reprogramming.. Nat Rev Mol Cell Biol.

[6] Yang X, Smith SL, Tian XC, Lewin HA, Renard JP (2007). Nuclear reprogramming of cloned embryos and its implications for therapeutic cloning.. Nat Genet.

[7] Greenstein JS, Murray RW, Foley RC (1958). Observations on the morphogenesis and histochemistry of the bovine preattachment placenta between 16 and 33 days of gestation.. Anat Rec.

[8] Heyman Y (2005). Nuclear transfer: a new tool for reproductive biotechnology in cattle.. Reprod Nutr Dev.

[9] King WA, Coppola G, Alexander B, Mastromonaco G, Perrault S (2006). The impact of chromosomal alteration on embryo development.. Theriogenology.

[10] Loi P, Beaujean N, Khochbin S, Fulka J, Ptak G (2008). Asymmetric nuclear reprogramming in somatic cell nuclear transfer?. Bioessays.

[11] Niemann H, Tian XC, King WA, Lee RS (2008). Epigenetic reprogramming in embryonic and foetal development upon somatic cell nuclear transfer cloning.. Reproduction.

[12] Tian XC, Kubota C, Enright B, Yang X (2003). Cloning animals by somatic cell nuclear transfer–biological factors.. Reprod Biol Endocrinol.

[13] Whitworth KM, Prather RS (2010). Somatic cell nuclear transfer efficiency: how can it be improved through nuclear remodeling and reprogramming?. Mol Reprod Dev.

[14] Wilmut I (2006). Are there any normal clones?. Methods Mol Biol.

[15] Zhao J, Whyte J, Prather RS (2010). Effect of epigenetic regulation during swine embryogenesis and on cloning by nuclear transfer.. Cell Tissue Res.

[16] Berg DK, van Leeuwen J, Beaumont S, Berg M, Pfeffer PL (2010). Embryo loss in cattle between Days 7 and 16 of pregnancy.. Theriogenology.

[17] Kato Y, Li X, Amarnath D, Ushizawa K, Hashizume K (2007). Comparative gene expression analysis of bovine nuclear-transferred embryos with different developmental potential by cDNA microarray and real-time PCR to determine genes that might reflect calf normality.. Cloning Stem Cells.

[18] Lucifero D, Suzuki J, Bordignon V, Martel J, Vigneault C (2006). Bovine SNRPN methylation imprint in oocytes and day 17 in vitro-produced and somatic cell nuclear transfer embryos.. Biol Reprod.

[19] Rodriguez-Alvarez L, Sharbati J, Sharbati S, Cox JF, Einspanier R (2010). Differential gene expression in bovine elongated (Day 17) embryos produced by somatic cell nucleus transfer and in vitro fertilization.. Theriogenology.

[20] Suzuki J, Therrien J, Filion F, Lefebvre R, Goff AK (2011). Loss of methylation at H19 DMD is associated with biallelic expression and reduced development in cattle derived by somatic cell nuclear transfer.. Biol Reprod.

[21] Heyman Y, Camous S, Fevre J, Meziou W, Martal J (1984). Maintenance of the corpus luteum after uterine transfer of trophoblastic vesicles to cyclic cows and ewes.. J Reprod Fertil.

[22] Alexopoulos NI, Maddox-Hyttel P, Tveden-Nyborg P, D’Cruz NT, Tecirlioglu TR (2008). Developmental disparity between in vitro-produced and somatic cell nuclear transfer bovine days 14 and 21 embryos: implications for embryonic loss.. Reproduction.

[23] Degrelle SA, Campion E, Cabau C, Piumi F, Reinaud P (2005). Molecular evidence for a critical period in mural trophoblast development in bovine blastocysts.. Dev Biol.

[24] Degrelle SA, Le Cao KA, Heyman Y, Everts RE, Campion E (2011). A small set of extra-embryonic genes defines a new landmark for bovine embryo staging.. Reproduction.

[25] Betteridge K, Flechon J (1988). The anatomy and physiology of pre-attachment bovine embryos.. Theriogenology.

[26] Hue I, Degrelle SA, Campion E, Renard JP (2007). Gene expression in elongating and gastrulating embryos from ruminants.. Soc Reprod.

[27] Maddox-Hyttel P, Alexopoulos NI, Vajta G, Lewis I, Rogers P (2003). Immunohistochemical and ultrastructural characterization of the initial post-hatching development of bovine embryos.. Reproduction.

[28] Flechon JE, Degrouard J, Flechon B (2004). Gastrulation events in the prestreak pig embryo: ultrastructure and cell markers.. Genesis.

[29] Guillomot M (1995). Cellular interactions during implantation in domestic ruminants.. J Reprod.

[30] Flechon JE, Flechon B, Degrouard J, Guillomot M (2007). Cellular features of the extra-embryonic endoderm during elongation in the ovine conceptus.. Genesis.

[31] Guillomot M, Turbe A, Hue I, Renard JP (2004). Staging of ovine embryos and expression of the T-box genes Brachyury and Eomesodermin around gastrulation.. Reproduction.

[32] Bui LC, Evsikov AV, Khan DR, Archilla C, Peynot N (2009). Retrotransposon expression as a defining event of genome reprogramming in fertilized and cloned bovine embryos.. Reproduction.

[33] Renard JP, Maruotti J, Jouneau A, Vignon X (2007). Nuclear reprogramming and pluripotency of embryonic cells: Application to the isolation of embryonic stem cells in farm animals.. Theriogenology.

[34] Ameen N, Apodaca G (2007). Defective CFTR apical endocytosis and enterocyte brush border in myosin VI-deficient mice.. Traffic.

[35] Longo-Guess CM, Gagnon LH, Cook SA, Wu J, Zheng QY (2005). A missense mutation in the previously undescribed gene Tmhs underlies deafness in hurry-scurry (hscy) mice.. Proc Natl Acad Sci U S A.

[36] Self T, Sobe T, Copeland NG, Jenkins NA, Avraham KB (1999). Role of myosin VI in the differentiation of cochlear hair cells.. Dev Biol.

[37] Nadra K, Anghel SI, Joye E, Tan NS, Basu-Modak S (2006). Differentiation of trophoblast giant cells and their metabolic functions are dependent on peroxisome proliferator-activated receptor beta/delta.. Mol Cell Biol.

[38] Hue I, Renard JP, Viebahn C (2001). Brachyury is expressed in gastrulating bovine embryos well ahead of implantation.. Dev Genes Evol.

[39] Martin L, Besch-Williford C, Lai L, Cheong HT, Im GS (2007). Morphologic and histologic comparisons between in vivo and nuclear transfer derived porcine embryos.. Mol Reprod Dev.

[40] Rodriguez-Alvarez L, Cox J, Tovar H, Einspanier R, Castro FO (2010). Changes in the expression of pluripotency-associated genes during preimplantation and peri-implantation stages in bovine cloned and in vitro produced embryos.. Zygote.

[41] Tveden-Nyborg P, Peura TT, Hartwich KM, Walker SK, Maddox-Hyttel P (2005). Morphological characterization of pre- and peri-implantation in vitro cultured, somatic cell nuclear transfer and in vivo derived ovine embryos.. Reproduction.

[42] Hasler JF, Henderson WB, Hurtgen PJ, Jin ZQ, McCauley AD (1995). Production, freezing and transfer of bovine IVF embryos and subsequent calving results.. Theriogenology.

[43] Reichenbach HD, Liebrich J, Berg U, Brem G (1992). Pregnancy rates and births after unilateral or bilateral transfer of bovine embryos produced in vitro.. J Reprod Fertil.

[44] Chesne P, Adenot PG, Viglietta C, Baratte M, Boulanger L (2002). Cloned rabbits produced by nuclear transfer from adult somatic cells.. Nat Biotechnol.

[45] Bauersachs S, Ulbrich SE, Zakhartchenko V, Minten M, Reichenbach M (2009). The endometrium responds differently to cloned versus fertilized embryos.. Proc Natl Acad Sci U S A.

[46] Mansouri-Attia N, Sandra O, Aubert J, Degrelle S, Everts RE (2009). Endometrium as an early sensor of in vitro embryo manipulation technologies.. Proc Natl Acad Sci U S A.

[47] Bertocchini F, Stern CD (2002). The hypoblast of the chick embryo positions the primitive streak by antagonizing nodal signaling.. Dev Cell.

[48] Idkowiak J, Weisheit G, Plitzner J, Viebahn C (2004). Hypoblast controls mesoderm generation and axial patterning in the gastrulating rabbit embryo.. Dev Genes Evol.

[49] Perea-Gomez A, Vella FD, Shawlot W, Oulad-Abdelghani M, Chazaud C (2002). Nodal antagonists in the anterior visceral endoderm prevent the formation of multiple primitive streaks.. Dev Cell.

[50] Jouneau A, Zhou Q, Camus A, Brochard V, Maulny L (2006). Developmental abnormalities of NT mouse embryos appear early after implantation.. Development.

[51] Adams HA, Southey BR, Everts RE, Marjani SL, Tian CX (2011). Transferase activity function and system development process are critical in cattle embryo development.. Funct Integr Genomics.

[52] Maruotti J, Muñoz M, Degrelle SA, Gómez E, Louet C (2012). Efficient derivation of bovine embryonic stem cells needs more than active core pluripotency factors..

[53] Alberio R, Croxall N, Allegrucci C (2010). Pig epiblast stem cells depend on activin/nodal signaling for pluripotency and self-renewal.. Stem Cells Dev.

[54] Maruotti J, Dai XP, Brochard V, Jouneau L, Liu J (2010). Nuclear transfer-derived epiblast stem cells are transcriptionally and epigenetically distinguishable from their fertilized-derived counterparts.. Stem Cells.

[55] Sawai K (2009). Studies on gene expression in bovine embryos derived from somatic cell nuclear transfer.. J Reprod Dev.

[56] Sawai K, Kageyama S, Moriyasu S, Hirayama H, Minamihashi A (2007). Changes in the mRNA transcripts of insulin-like growth factor ligand, receptors and binding proteins in bovine blastocysts and elongated embryos derived from somatic cell nuclear transfer.. J Reprod Dev.

[57] Fujii T, Moriyasu S, Hirayama H, Hashizume T, Sawai K (2010). Aberrant expression patterns of genes involved in segregation of inner cell mass and trophectoderm lineages in bovine embryos derived from somatic cell nuclear transfer.. Cell Reprogram.

[58] Smith CS, Berg DK, Berg M, Pfeffer PL (2010). Nuclear transfer-specific defects are not apparent during the second week of embryogenesis in cattle.. Cell Reprogram.

[59] Kim JY, Burghardt RC, Wu G, Johnson GA, Spencer TE (2011). Select nutrients in the ovine uterine lumen. VII. Effects of arginine, leucine, glutamine, and glucose on trophectoderm cell signaling, proliferation, and migration.. Biol Reprod.

[60] Rozario T, Dzamba B, Weber GF, Davidson LA, DeSimone DW (2009). The physical state of fibronectin matrix differentially regulates morphogenetic movements in vivo.. Dev Biol.

[61] Miki H, Wakisaka N, Inoue K, Ogonuki N, Mori M (2009). Embryonic rather than extraembryonic tissues have more impact on the development of placental hyperplasia in cloned mice.. Placenta.

[62] Soares MJ, Asanoma K (2009). Trophoblast stem cells derived from nuclear transfer embryos: phenotypically unique, bad neighbors, or poor communicators?. Proc Natl Acad Sci U S A.

[63] Chavatte-Palmer P, Camous S, Jammes H, Le Cleac’h N, Guillomot M (2011). Review: Placental perturbations induce the developmental abnormalities often observed in bovine somatic cell nuclear transfer..

[64] Schuettengruber B, Cavalli G (2009). Recruitment of polycomb group complexes and their role in the dynamic regulation of cell fate choice.. Development.

[65] Fitzpatrick E, Johnson MP, Dyer TD, Forrest S, Elliott K (2009). Genetic association of the activin A receptor gene (ACVR2A) and pre-eclampsia.. Mol Hum Reprod.

[66] Roten LT, Johnson MP, Forsmo S, Fitzpatrick E, Dyer TD (2009). Association between the candidate susceptibility gene ACVR2A on chromosome 2q22 and pre-eclampsia in a large Norwegian population-based study (the HUNT study).. Eur J Hum Genet.

[67] van Dijk M, Oudejans CB (2011). STOX1: Key Player in Trophoblast Dysfunction Underlying Early Onset Preeclampsia with Growth Retardation..

[68] Takahashi K, Yamanaka S (2006). Induction of pluripotent stem cells from mouse embryonic and adult fibroblast cultures by defined factors.. Cell.

[69] Wernig M, Meissner A, Foreman R, Brambrink T, Ku M (2007). In vitro reprogramming of fibroblasts into a pluripotent ES-cell-like state.. Nature.

[70] Yu F, Li J, Chen H, Fu J, Ray S (2011). Kruppel-like factor 4 (KLF4) is required for maintenance of breast cancer stem cells and for cell migration and invasion.. Oncogene.

[71] Akaogi K, Nakajima Y, Ito I, Kawasaki S, Oie SH (2009). KLF4 suppresses estrogen-dependent breast cancer growth by inhibiting the transcriptional activity of ERalpha.. Oncogene.

[72] Kee HJ, Kook H (2009). Kruppel-like factor 4 mediates histone deacetylase inhibitor-induced prevention of cardiac hypertrophy.. J Mol Cell Cardiol.

[73] Khan DR, Dube D, Gall L, Peynot N, Ruffini S (2012). Expression of Pluripotency Master Regulators during Two Key Developmental Transitions: EGA and Early Lineage Specification in the Bovine Embryo.. PLoS One.

[74] Dai X, Hao J, Hou XJ, Hai T, Fan Y (2010). Somatic nucleus reprogramming is significantly improved by m-carboxycinnamic acid bishydroxamide, a histone deacetylase inhibitor.. J Biol Chem.

[75] Hai T, Hao J, Wang L, Jouneau A, Zhou Q (2011). Pluripotency maintenance in mouse somatic cell nuclear transfer embryos and its improvement by treatment with the histone deacetylase inhibitor TSA.. Cell Reprogram.

[76] Matoba S, Inoue K, Kohda T, Sugimoto M, Mizutani E (2011). RNAi-mediated knockdown of Xist can rescue the impaired postimplantation development of cloned mouse embryos.. Proc Natl Acad Sci U S A.

[77] Vignon X, Le Bourhis D, Adenot P, Marchal J, Lavergne Y (2000). Production of bovine embryos by nuclear transfer of adult skin fibroblasts: the effect of serum starvation.. Theriogenology.

[78] Alves BC, Hossepian de Lima VF, Teixeira CM, Moreira-Filho CA (2003). Use of primers derived from a new sequence of the bovine Y chromosome for sexing Bos taurus and Bos indicus embryos.. Theriogenology.

[79] Degrelle SA, Hennequet-Antier C, Chiapello H, Piot-Kaminski K, Piumi F (2008). Amplification biases: possible differences among deviating gene expressions.. BMC Genomics.

[80] Everts RE, Band MR, Liu ZL, Kumar CG, Liu L (2005). A 7872 cDNA microarray and its use in bovine functional genomics.. Vet Immunol Immunopathol.

[81] Jaffrezic F, Marot G, Degrelle S, Hue I, Foulley JL (2007). A structural mixed model for variances in differential gene expression studies.. Genet Res.

[82] Degrelle SA, Murthi P, Evain-Brion D, Fournier T, Hue I (2011). Expression and localization of DLX3, PPARG and SP1 in bovine trophoblast during binucleated cell differentiation.. Placenta.

[83] Ushizawa K, Takahashi T, Hosoe M, Kizaki K, Hashizume K (2009). Characterization and expression analysis of SOLD1, a novel member of the retrotransposon-derived Ly-6 superfamily, in bovine placental villi.. PLoS One.

[84] Ushizawa K, Takahashi T, Hosoe M, Kizaki K, Hashizume K (2010). Cloning and expression of SOLD1 in ovine and caprine placenta, and their expected roles during the development of placentomes.. BMC Dev Biol.

