# Supplementary figures and images for: Uncoupled Embryonic and Extra-Embryonic Tissues Compromise Blastocyst Development after Somatic Cell Nuclear Transfer
Source: PLoS One. 2012 Jun 6;7(6):e38309. doi: 10.1371/journal.pone.0038309 (PMC3368877; doi:10.1371/journal.pone.0038309)

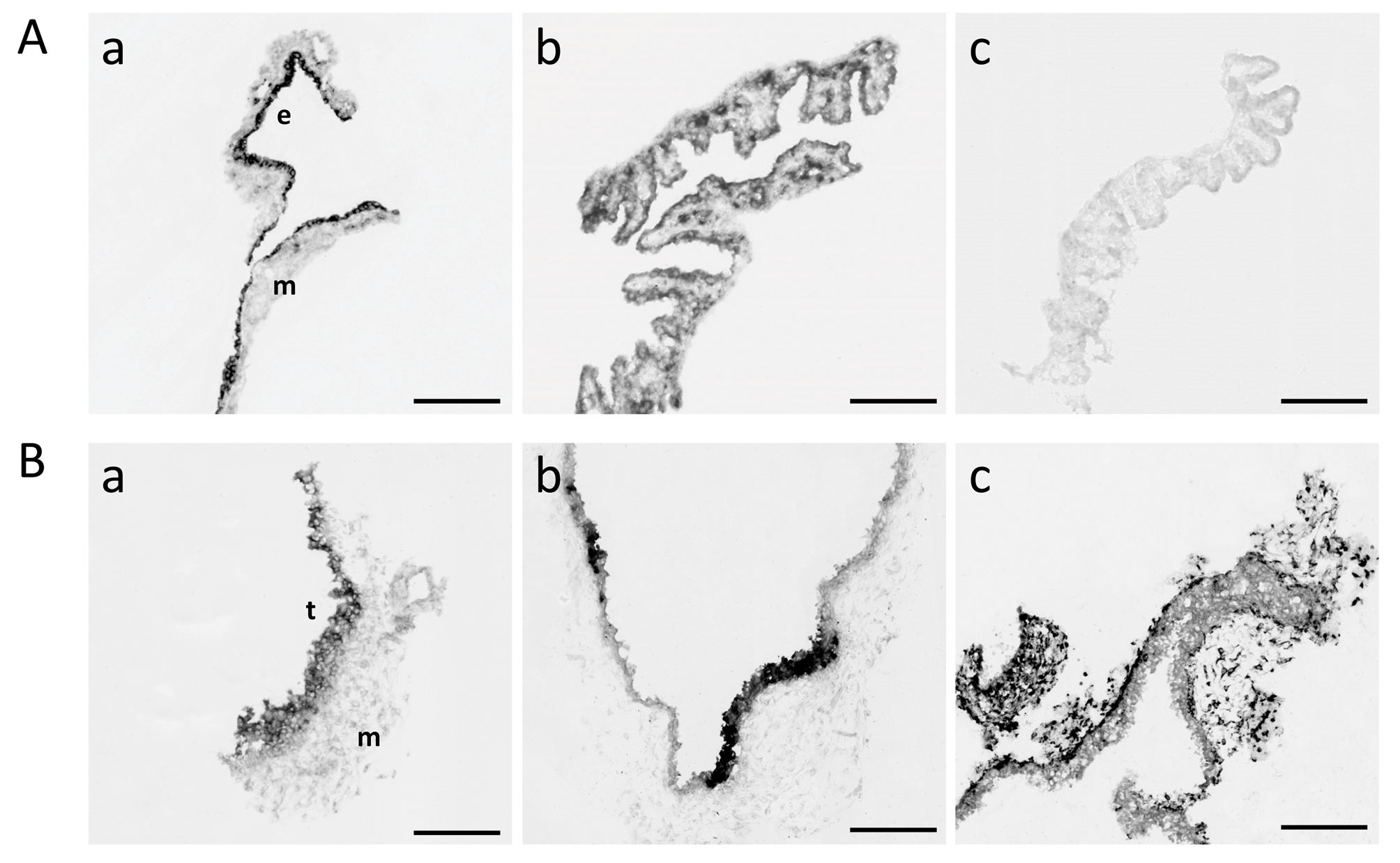

Supplement: Figure S1 — Additional ISH on AI samples from Days 25 to 63. A) The yolk sac at D25 (a) or D36 (b, c) is composed of endoderm (e) and mesoderm (m). For example, FN1, FGA, U2AF2, DUSP14, ANKRD33 were expressed in the endoderm at these stages. Here, only FN1 is illustrated (a, b). Other genes like B4GALT1, PAG11 or TKDP3 were absent from the yolk sac (as in c). B) The chorion at D63 (a-c) is composed of trophoblast (t) and mesoderm (m). Most of the genes tested were expressed in the trophoblast as in (a), namely B4GALT1, DFFA, PAG11, TADA3, TUB1A1. Only a few showed a different pattern, as illustrated by TKDP3 in the trophoblast (b) or COL1A2 in the mesoderm (c). Another example is provided by PLIN2 in Fig. 3. (TIF) [file pone.0038309.s001.tif]
