# Supplementary material for: Uncoupled Embryonic and Extra-Embryonic Tissues Compromise Blastocyst Development after Somatic Cell Nuclear Transfer
Source: PLoS One. 2012 Jun 6;7(6):e38309. doi: 10.1371/journal.pone.0038309 (PMC3368877; doi:10.1371/journal.pone.0038309)
Supplement: Table S1 — Statistical analyses. (DOC) [file pone.0038309.s002.doc]

| **Analyses** | **Tissue**  (EET) | **Conditions** | **Test**  **(FDR)** | **Differential**  **EST** | **Discriminative**  **EST** |
| --- | --- | --- | --- | --- | --- |
| Differential  Analyses  Pairwise  Comparisons  Using  SMVar | n=10 | Intra AI | 10% | 0 | - |
|  |  | Intra IVP |  | 0 | - |
|  |  | Intra SCNT High |  | 0 | - |
|  |  | Intra SCNT Med |  | 0 | - |
|  |  | Intra SCNT Low |  | 0 | - |
|  | n=20 | Male-Female among controls (AI+IVP) | 20% | 0 | - |
|  | n=40 | Female : SCNT-AI-IVP (n=30/6/4) |  | 0 | - |
|  | n=50 | Pregnant recipients (100 cows used for transfers or AI ;  38 pregnant cows) |  | 2*  *not included in the above DEG lists | - |
|  | n=60 | Pregnant Breeds (9) |  | 0 | - |
|  | n=18 | Normal SCNT (embryonic stages:  N1-N2-D) |  | 0 | - |
|  | n=28 | Normal-Abnormal SCNT (embryonic stages :  Ab1+Ab2 versus  N1-N2-D) |  | 0 | - |
| Classifying  Methods :  CART, SVM,  RF | n=21 | Normal-Abnormal SCNT (embryonic stages  (Ab1+Ab2 versus N2-D) | - | - | 2*  *common to the 3 classification methods |

Table S1: statistical analyses
