# Supplementary material for: Uncoupled Embryonic and Extra-Embryonic Tissues Compromise Blastocyst Development after Somatic Cell Nuclear Transfer
Source: PLoS One. 2012 Jun 6;7(6):e38309. doi: 10.1371/journal.pone.0038309 (PMC3368877; doi:10.1371/journal.pone.0038309)
Supplement: Table S2 — Primers for QPCR. (DOC) [file pone.0038309.s003.doc]

Table S1: primers for QPCR

| **EST Acc Numb** | **Gene ID** | **primer forward** | **primer reverse** | **Amplicon size in bp** |
| --- | --- | --- | --- | --- |
| AW463989 | *MYO6* | CTCGCTGTCCGTCATCAAATT | TTTTCAGTGTGCCCACCTTG | 146 |
| AW465534 | *FN1* | GCGATTGTCTGATTCTGGCTT | TCTCCCTGACGATCCCACTT | 141 |
| BF040851 | *ALG5* | TGCCACAGCTTCATCGACAT | GGCAACCGTTTTTCTTCATTG | 142 |
| BF041292 | *LHFPL2* | TTACTGGCGTTGGTCCTGGAT | TGCTAGGAGCTTGGTTGAGCA | 140 |
| BF043574 | *EIF2S3* | TGGAACAAAAATTGACCCCAC | TGTGCGTACACCAAGAAGTCG | 142 |
| BF043749 | *PLIN2* | GGTGACCGAGTCTGAGAGTGC | GCAGGTGTCAGCTCAAGAGGA | 142 |
| BF045264 | *DFFA* | GGAAACCTGGCATCTCAGCA | TAGGGTCCTCCTTGGCAACC | 141 |
| BF045665 | *CCNE1* | AGAATGAGGCTCTGATTGGCA | GTGCTCGGGAAGAAATCACAC | 142 |
| BF046406 | *APLP2* | AGCTTTTGCGCTTCAGCATC | AGCTACGGTGAGTCAATGGCA | 149 |
| CN432421 | *C5orf13* | TCAGCATTGTAGGTTGAGCGA | GGCGAGGCTACTGAAAGGAAG | 147 |
| CN433942 | *PGAM5* | TCTCCCCTACCCCTTGTTGG | AAGCCCCTGGTCAGACTTCC | 131 |
| CN434085 | *BIN3* | CAGAAACCCGCAGACAGGTAA | ATGTGGTCCCAACTGTGCAG | 138 |
| CN435723 | *WNT5B* | TGGACTACGGCTACCGCTTC | TTTGCAGGTTCATGAGCACG | 108 |
| CR552847 | *MSH3* | ATGATGGAATCGCCATTGCT | ATTCCCCACCTGCTGTGAGTA | 131 |
| CR552898 | *ASF1A* | GAGCTCGGGTACAGCCAATC | GCGGCGACTTGAGAAACTTTT | 146 |
| CR552963 | *EED* | CAAATGCACAACGCTGACTCA | TTTATCGCAGTCGGTCCCAG | 131 |
|  |  |  |  |  |
|  | *GAPDH* | CACTACCATGGAGAAGGCTGG | GTGGTTCACGCCCATCACA | 106 |
|  | *ACTIN* | GCTTTACCACCACAGCCGAG | CGACGCAGCAGTAGCCATCT | 100 |
|  | *RPL19* | GAAAGTCTGGTTGGACCCCA | CCGGGAATGGACAGTCACAG | 121 |
|  | *RPS18* | CACGCCAATACAAGATCCCA | AGGCGCTCCAGGTCTTCAC | 121 |
