# Supplementary material for: Uncoupled Embryonic and Extra-Embryonic Tissues Compromise Blastocyst Development after Somatic Cell Nuclear Transfer
Source: PLoS One. 2012 Jun 6;7(6):e38309. doi: 10.1371/journal.pone.0038309 (PMC3368877; doi:10.1371/journal.pone.0038309)
Supplement: Table S3 — Primers for DNA templates before in vitro transcription. (DOC) [file pone.0038309.s004.doc]

Table S3: Primers for DNA templates before in vitro transcription

| **Genbank Acc Nb** | **PLASMID** | **RIBOPROBE** | | **PRIMERS for DNA template** |
| --- | --- | --- | --- | --- |
| AW…, BF… | pT3T7Pac | SENSE | T7  RNA pol | M13R :  5’-CACAGGAAACAGCTATGACC-3’ |
|  |  |  |  | Bq7KF :  5’-TGCTTGCGGCCGCATTTGTTT-3’ |
|  |  | ANTI- SENSE | T3  RNA pol | M13F :  5’-GTAAAACGACGGCCAGTGA-3’ |
|  |  |  |  | Bq7KR :  5’-GCCCTCGAGGCCAAGAAT-3’ |
| CR…, CN…, CV… | pGEMZf11(+) | SENSE | T7  RNA pol | M13F :  5’-GTAAAACGACGGCCAGTGA-3’ |
|  |  |  |  | BqUSR :  5’-CTCAAGCTTATGCATGCGG-3’ |
|  |  | ANTI- SENSE | Sp6  RNA pol | M13R :  5’-CACAGGAAACAGCTATGACC-3’ |
|  |  |  |  | BqUSF :  5’-ATTGGCCAAGTCGGCCGA-3’ |
